# siRNA Screening of a Targeted Library of DNA Repair Factors in HIV Infection Reveals a Role for Base Excision Repair in HIV Integration

**DOI:** 10.1371/journal.pone.0017612

**Published:** 2011-03-23

**Authors:** Amy S. Espeseth, Rick Fishel, Daria Hazuda, Qian Huang, Min Xu, Kristine Yoder, Honglin Zhou

**Affiliations:** 1 Department of Antiviral Research, Merck Research Laboratories, West Point, Pennsylvania, United States of America; 2 Department of Molecular Virology, Immunology, and Medical Genetics, The Ohio State University Medical Center, Columbus, Ohio, United States of America; University of Minnesota, United States of America

## Abstract

Host DNA repair enzymes have long been assumed to play a role in HIV replication, and many different DNA repair factors have been associated with HIV. In order to identify DNA repair pathways required for HIV infection, we conducted a targeted siRNA screen using 232 siRNA pools for genes associated with DNA repair. Mapping the genes targeted by effective siRNA pools to well-defined DNA repair pathways revealed that many of the siRNAs targeting enzymes associated with the short patch base excision repair (BER) pathway reduced HIV infection. For six siRNA pools targeting BER enzymes, the negative effect of mRNA knockdown was rescued by expression of the corresponding cDNA, validating the importance of the gene in HIV replication. Additionally, mouse embryo fibroblasts (MEFs) lacking expression of specific BER enzymes had decreased transduction by HIV-based retroviral vectors. Examining the role BER enzymes play in HIV infection suggests a role for the BER pathway in HIV integration.

## Introduction

The retroviral life cycle requires that viral proteins co-opt host factors to support virus production. Following HIV entry which is initiated by the virus binding to the CD4 and either CXCR4 or CCR5 co-receptors, the viral capsid enters the cytoplasm, viral RNA is then uncoated and reverse transcribed, and the reverse transcribed viral DNA is imported to the nucleus and integrated into the host genome. The processes of reverse transcription and integration are likely to require host DNA repair pathways at various steps. Reverse transcription is discontinuous; primers must be excised and discontinuities in the viral DNA must be repaired [1]. Integration of the viral DNA into host chromatin produces a gapped intermediate with unjoined viral 5′ ends [2]. In both cases, these gaps and discontinuities have long been assumed to be repaired by host DNA repair pathways, but the nature of these pathways has remained elusive.

The discovery of RNA interference has allowed loss of function phenotypes for large numbers of genes to be screened in a single experiment. With this technology, arrays of double stranded, 19–21 nt RNAs can be designed to knock down the mRNA level of a targeted gene, allowing a rapid assessment of the effect of a loss of gene function on a specific cell phenotype following siRNA transfection [3], [4].

Recently, a number of genome scale siRNA screens were conducted that together identified over 1000 different host factors associated with HIV replication, including a number of DNA repair factors [5]–[7]. Reasoning that a smaller scale, targeted screen might provide better focus on specific pathways of interest, we screened an siRNA library targeting DNA repair genes for effects on HIV replication. We identified a number of genes involved in the short patch Base Excision Repair (BER) pathway, a DNA repair pathway responsible for repairing damage triggered by oxidation or alkylation of single nucleotides [8].

## Results

### Screening of an siRNA library targeting DNA repair genes reveals the importance of BER factors in HIV infection

**Figure 1:**
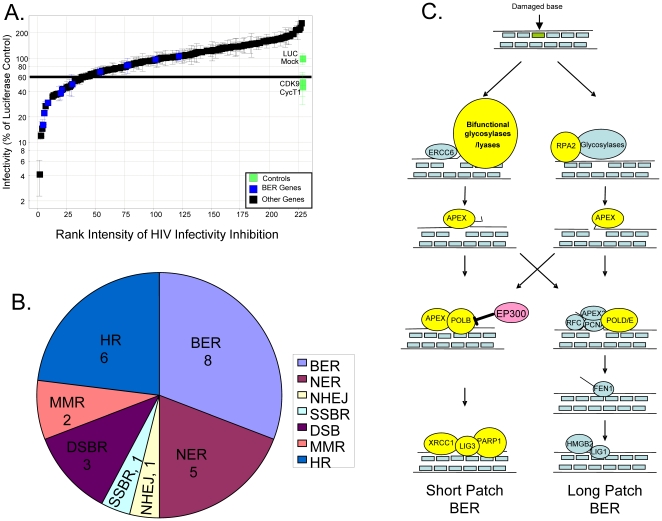
siRNA screen for DNA repair factors associated with HIV infection revealed a role for the BER pathway. *(A)* Data from siRNA screen represented relative to the negative control (N = 2). The green squares represent control siRNAs. Negative controls were luciferase (nonsilencing siRNA) and mock (no siRNA). Positive controls were siRNAs targeting CDK9 and Cyclin T1 (CycT1). The black squares represent siRNAs that do not target the BER pathway. The blue squares represent siRNAs targeting genes in the BER pathway. The horizontal line running across the graph indicates 40% inhibition; siRNAs that resulted in less than 40% infectivity were considered to be hits in the screen. *(B)* DNA repair pathways targeted by effective siRNAs (BER, base excision repair; NER, nucleotide excision repair; NHEJ, non-homologous end joining; SSBR, single stranded break repair; DSB, double stranded break repair; MMR, mismatch repair; HR, homologous recombination). The number of hits assigned to each pathway is indicated. Of the 35 non-toxic siRNA pools that led to 40% or greater inhibition of HIV infectivity in the primary screen, 8 mapped to BER, 5 mapped to NER, 1 mapped to NHEJ, 1 mapped to SSBR, 3 mapped to DSB, 2 mapped to MMR, 6 mapped to HR. The remaining 9 could not be mapped to specific DNA repair pathways and are not included in the diagram. *(C)* Diagram of the BER pathway based on [13]. siRNAs targeting BER factors colored yellow decreased HIV infectivity by at least 40%, siRNAs targeting BER factors colored red increased HIV infectivity by at least 2×. siRNAs targeted BER factors colored blue had no effect on HIV infection.

To identify DNA repair mechanisms associated with retroviral infection, we transfected an siRNA library targeting 232 DNA repair genes (GO:0006281) (Table S1) into HeLa P4/R5 cells. siRNAs targeting Cyclin T1 and CDK9 were used as positive controls for inhibition of HIV infection, and an siRNA targeting luciferase and mock transfection as negative controls. The cells were infected with HIV HXB2 and assayed for β-galactosidase expression as a reporter for successful infection 48 h later [5]. The positive control siRNAs resulted in a 40 to 50% decrease (Figure 1A); thus, we elected to evaluate more fully all siRNAs that resulted in more than 40% inhibition of HIV infection.

After screening the library in duplicate, we identified 41 siRNA pools that lowered HIV infection. Six of these decreased cell viability and were not studied further. The remaining 35 siRNA targets were mapped to DNA repair pathways by GO annotation (Figure 1B, Table S2). Strikingly, the non-homologous end joining pathway (NHEJ), which has frequently been linked to HIV replication, was represented by only one of the hits in this screen. In contrast, 23% of the mapped genes were associated with the base-excision repair (BER) pathway (Figure 1B, Table S2) [9]–[12]. Since the largest number of mapped genes were assigned to the BER pathway, we elected to examine the role of this pathway in HIV infection further.

The results from the primary screen indicated that siRNAs targeting a number of genes in the BER pathway decrease HIV infection. To confirm the specificity of the siRNA pools identified in the screen and to control for off-target silencing, we evaluated the efficacy of the single siRNAs in each of the effective siRNA pools identified in the BER pathway, and found at least two effective single siRNAs targeting each of the BER genes identified in the screen. We then probed the pathway further, and evaluated the efficacy of single siRNAs targeting genes associated with BER that were not identified in the screen. We found that two or more separate single siRNAs targeting MUTYH, NTHL1, NEIL3, XRCC1, LIG3, and POLB were capable of reducing HIV infection by at least 40%, further implicating BER as a pathway important for HIV replication.

The BER pathway is outlined in Figure 1C (modeled as in ref [13]). BER is initiated when a DNA damage-sensing protein identifies a damaged base. BER may then proceed down one of two distinct mechanistic pathways termed “short patch” and “long patch” BER [14]. In both pathways, the damaged base is removed by a glycosylase, creating an abasic (AP) site. The glycosylase may be a monofunctional glycosylase, associated with both short and long patch repair, or a bifunctional glycosylase/β-lyase, associated with short patch repair only. Of the monofunctional glycosylases, only siRNAs targeting MUTYH decreased HIV infection. In contrast, siRNAs targeting six different bifunctional glycosylase/β-lyase enzymes inhibited HIV infection by at least 50% (Figure 1C and Table 1).

**Table 1:** Effect on HIV infectivity of siRNAs targeting BER-pathway genes.

*Base-Excision Repair Genes*	*Role in BER*	*Effect of siRNAs on HIV Infection (% of Control)*
APTX	Bridge	221
PARP1	Damage sensor	30
DDB2	DNA binding	30
RPA2	DNA binding	38
APEX1	Endonuclease	36
MBD4	Glycosylase	124
MPG	Glycosylase	84
TDG	Glycosylase	164
UNG	Glycosylase	138
UNG2	Glycosylase	131
MUTYH	Glycosylase	39
ERCC5	Short patch: promotes NTHL1 DNA binding	163
ERCC6	Helicase	74
FEN1	Long Patch	107
HMGB2	Long Patch	119
LIG1	Long Patch	119
PCNA	Long Patch	209
POLD1	Long Patch Polymerase	185
POLE	Long Patch Polymerase	36
APEX2	Long Patch Endonuclease	158
NEIL1	Short patch β-lyase	135
NEIL2	Short patch β-lyase	32
NEIL3	Short patch β-lyase	30
NTHL1	Short patch β-lyase	47
OGG1	Short patch β-lyase	45
POLI	Short patch β-lyase	16
POLL	Short patch β-lyase	22
EP300	Reduces POLB activity	264
LIG3	Short patch ligase	42
POLB	Short patch polymerase	42
XRCC1	Short patch–binds LIG3	45

Following cleavage of the phosphodiester bond 3′ to the AP site, the endonuclease APEX1 cleaves the phosphodiester bond 5′ to the site, liberating the 3′ sugar residue and creating a gap. siRNAs targeting APEX1 inhibited HIV infection by more than 60%. In the short patch BER process, APEX1 recruits POLB to fill in the single nucleotide gap, and the DNA backbone is then repaired by an enzymatic complex including LIG3 and XRCC1. siRNAs targeting POLB, LIG3, and XRCC1 inhibited HIV infection by more than 50%. Although long path BER enzymes have been used to model repair of HIV integration intermediates in vitro [15], siRNAs targeting these genes had little effect on HIV infection (Figure 1C and Table 1).

### Confirmation of the specificity of BER targeting siRNAs by cDNA rescue of the defect in HIV infection

We then validated the role of BER in HIV replication by demonstrating rescue of the siRNA-induced knockdown phenotype with expression of the targeted cDNA. siRNAs targeting the 3′ UTR of seven of the BER genes identified in our screen were evaluated for both mRNA knockdown and effects on HIV infectivity (Table 2). As shown in Table 2, we observed lowered mRNA levels and decreased HIV infectivity following transfection of at least two of the 3′UTR directed siRNAs targeting each of the seven BER genes, with the exception of OGG1, where, despite our ability to identify at least two effective siRNAs targeting the coding region of the mRNA, only one of the 3′UTR directed siRNAs reduced HIV infectivity by 50%.

**Table 2:** mRNA knockdown and effect on HIV infectivity of 3′UTR-directed siRNAs targeting BER-pathway genes.

*Targeted Gene*	*Targeted Sequence*	*mRNA Level (% of Control)*	*HIV Infectivity (% of Control)*
APEX1	**GCATTCTATTTCTCATGTA**	11	28
	ACCAGGCTCCTGTGATAGA	29	62
	ACAAAGACTACTAATGACT	37	89
LIG3	GGCAGATAGACACAGTATA	28	102
	CATACTCTCCTTTACCATA	23	54
	**CCTTTACCATACTACTGGA**	43	25
MUTYH	TGAGAATCCTGTTGTTAGT	48	43
	CCTGAGAATCCTGTTGTTA	51	34
	**GAGAATCCTGTTGTTAGTA**	39	26
NEIL2	ACGTCTAAGTGTCCAGAAA	11	113
	CTGCTTGTTTACTCCTTAA	10	13
	**GGTGCCAGTAGTATAATAT**	11	27
NTHL1	**GAAGTGGCTTTACGCTTCA**	65	36
	AAGCCACGCCTGTTGAATA	15	48
	GAAGCCACGCCTGTTGAAT	24	54
OGG1	**GTATGCTCATTGAACTTTA**	52	50
	TAATTCGAGTCATGGCTAA	56	150
	TTCGAGTCATGGCTAATTT	52	117
OGG1 (coding)	CAAAGTCCTGCACACTGGA	24	50
	GTTACCCTGGCTCAACTGT	29	67
POLL	**CCTGACCAACGCTCCAATA**	56	35
	TGACTAGCTTGGCCAGTAG	69	32
	TAGCTTGGCCAGTAGTCGA	73	50

**Figure 2:**
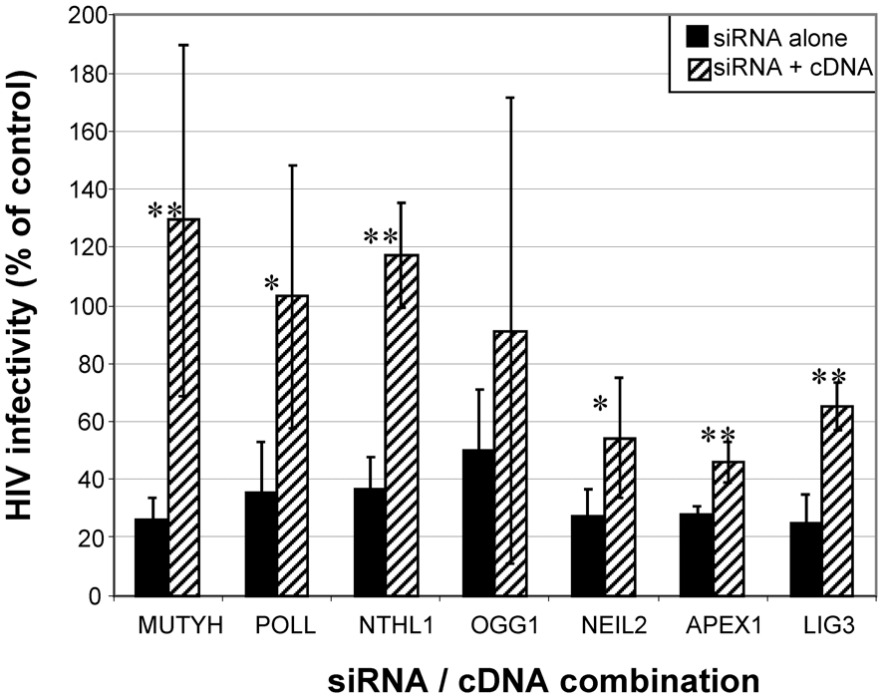
Effect of 3′UTR targeting siRNA on HIV infectivity and knockdown rescue by coexpression of cDNA. HeLa P4R5 cells were cotransfected with siRNAs targeting the 3′ UTR of the indicated gene and either an empty expression vector (grey bars) or a vector expressing the cDNA for the targeted gene (striped bars). N = 3, error bars indicate standard deviation. **p<0.01, *p<0.05 comparing empty vector and coexpressed cDNA.

The sequence of siRNAs directed against the 3′UTR of six different BER enzymes is shown, along with the amount of mRNA knockdown achieved in HeLa P4/R5 cells and the relative level of HIV infection in a single cycle assay relative to transfection of a nonsilencing siRNA following transfection of the siRNA. siRNAs in bold were used in the cDNA rescue experiments shown in Fig. 2.

Effective siRNAs were co-transfected into HeLa P4/R5 cells along with either an empty expression vector, or the vector expressing a cDNA for the targeted gene lacking the 3′UTR which was thus not affected by the introduction of the siRNA (Figure 2). We observed a significant increase in HIV infectivity upon cDNA rescue of siRNA knockdown for MUTYH, NTHL1, LIG3 and APEX1 (p<0.01), as well as with NEIL2 and POLL (p<0.05). Rescue with OGG1 did not reach statistical significance. Additionally, we earlier demonstrated that siRNAs targeting the 3′UTR of the β-lyase NEIL3 could effectively knock down NEIL3 mRNA levels and HIV infection of HeLa P4/R5 cells, and that the decrease in HIV infection could be rescued by NEIL3 cDNA expression [5]. Overall, the rescue of HIV infection by eight different BER cDNAs suggests a role for BER in HIV infection.

### Knockout of BER enzymes reduces HIV infection

**Figure 3:**
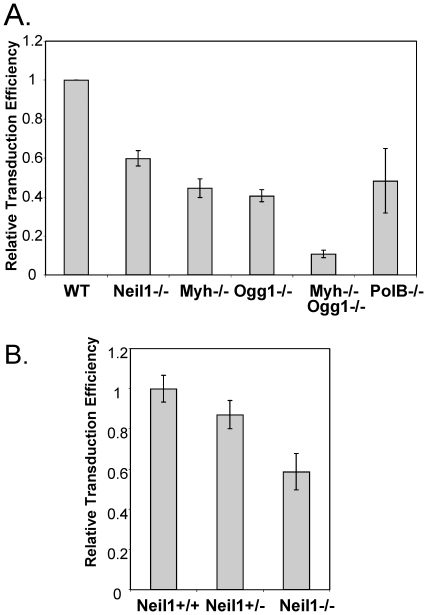
MEFs lacking BER genes have decreased transduction by HIV-based retroviral vectors. MEFs derived from mice with deletions of the *Neil1*, *Myh*, *Ogg1*, *Polβ*, or a double deletion of *Myh* and *Ogg1* genes. Cells were transduced with an HIV retroviral vector expressing GFP and analyzed at 72 hours post-infection by flow cytometry for GFP expression indicating successful infection. The percentage of GFP positive cells is shown relative to wild type MEFs from matched littermates. *(A)* Wild type, Neil1−/−, Myh−/−, Ogg1−/−, Myh−/− Ogg1−/−, and Polβ −/− (PolB−/−) cell lines, *(B)* Wild type (Neil1+/+), heterozygous (Neil1+/−), and Neil1−/− cell lines. Infections were performed at two MOI in duplicate at least three times. Error bars indicate the standard deviation after normalization.

The results of the siRNA screen were confirmed and extended with murine embryonic fibroblast cell lines (MEFs) from genetically defined BER deletion mice [16]–[19] (Figure 3). Wild type and BER null MEFs were infected with an HIV based retroviral vector expressing GFP following integration. Similar to the siRNA experiments, deletion of MUTYH, OGG1, and POLB led to decreased HIV infection (Figure 3A). In addition, infection of NEIL1 null MEFs indicates that this glycosylase plays a similar role in the HIV life cycle (Figures 3A and B). Expression of one allele at least partially restored HIV infection efficiency in NEIL1 heterozygous cells (Figure 3B). Cells with a complete deletion of OGG1 and MUTYH had less HIV infection than either heteroallelic deletion of OGG1 or MUTYH, indicating that these BER genes play non-redundant roles in the HIV life cycle. The decreased infection of BER null MEFs with an HIV-based retroviral vector supports the observations from the siRNA screen linking BER to HIV replication.

### BER is important for HIV vDNA integration

We sought to understand the requirement for BER within the HIV life cycle by evaluating HIV entry, reverse transcription, and transcription in HeLa P4/R5 cells transfected with siRNAs targeting BER genes. Assays for HIV entry, tat-mediated transcription, and reverse transcription were carried out as described [20], [5], [21]. siRNAs targeting CD4 and CXCR4 were included as positive controls in the HIV entry assay and decreased the entry of virus-like particles 79 and 67%, respectively. An siRNA targeting Cyclin T1 was included as a positive control in the Tat-mediated transcription assay, and knocked down tat-mediated transactivation of the HeLa P4/R5 LTR- βGAL reporter construct by 64%. As shown in Table 3, we did not observe consistent effects on viral entry, tat-mediated transcription, or reverse transcription following HIV infection of HeLa P4/R5 cells transfected with siRNAs targeting any of the BER enzymes assessed (Table 3).

**Table 3:** Effect of siRNAs targeting BER enzymes on HIV entry, reverse transcription, and tat-mediated transcription.

*Targeted Gene*	*Viral Entry (% Inhibition)*	*Reverse Transcription (% Inhibition)*	*Tat-mediated Transcription (% Inhibition)*
APEX1	9±4	ND	12±3
LIG3	5±4	ND	12±3
MUTYH	3±2	42±38	14±6
NEIL1	1±2	ND	19±5
NEIL2	10±3	ND	12±7
NEIL3	4±2	8±32	14±3
NTHL1	9±4	22±26	13±5
OGG1	6±3	ND	14±7
POLB	6±2	4±8	18±2
POLL	2±5	ND	16±3
CD4	79±1	ND	16±5
CXCR4	67±2	ND	21±2
Cyclin T1	ND	ND	64±4
LUC	0	0	0

**Figure 4:**
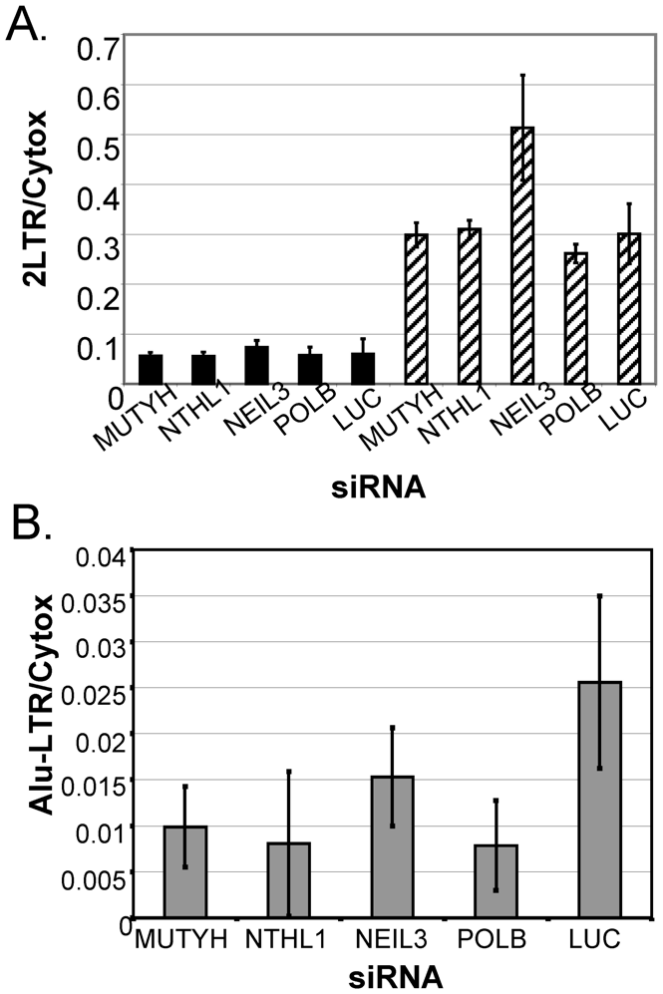
Knockdown of BER genes has no effect on nuclear localization, but does decrease HIV integration. *(A)*. Effect on 2LTR circle formation. HeLa P4/R5 cells were transfected with the indicated siRNAs and treated with the integrase inhibitor L-870,810 to increase 2LTR circle formation (striped bars) or left untreated (solid bars), and infected with HIV-1. 2LTR circles were measured as in (21). *(B)*. Effect of BER gene knockdown on HIV integration. HeLa P4/R5 cells were transfected with siRNAs targeting BER genes or a negative control siRNA targeting Luciferase. Cells were infected with HIV-1, DNA was isolated, and Alu-LTR PCR was conducted to determine the relative amount of integrated HIV-1 vDNA.

Following reverse transcription, HIV vDNA is transported to the nucleus where it is integrated into host DNA. 2LTR circles are the products of abortive retroviral integration efforts within the nucleus, and the production of these circles can be increased by treating HIV-infected cells with integrase inhibitors [22]. We found that transfecting HeLa P4/R5 cells with siRNAs targeting MUTYH, NTHL1, NEIL3 or POLB did not decrease 2LTR circle formation, suggesting that these genes may not play a role in nuclear localization of HIV DNA or in 2LTR circle formation (Figure 4A).

Once the vDNA has entered the nucleus, it is integrated into host chromosomal DNA. We measured the relative level of integrated vDNA in cells treated with siRNAs targeting BER genes using Alu-LTR qPCR [21]. HeLa P4/R5 cells transfected with siRNAs targeting MUTYH, NTHL1, NEIL3, or POLB consistently had 40–70% less integrated HIV DNA than cells transfected with LUC siRNA (Figure 4B). Expression of BER pathway enzymes thus appears to be required for successful completion of HIV vDNA integration.

## Discussion

We and others have shown that genome scale siRNA screens can identify hundreds of host factors necessary for robust HIV replication [5]–[7]. Here, we elected to carry out a focused siRNA screen to address the specific question of which DNA repair pathways are required for HIV replication. The use of a small siRNA library targeting a particular cellular function allowed for screening in duplicate, more in depth follow up of effective siRNAs, and the use of bioinformatic tools to identify pathways over-represented in the pool of hits.

**Figure 5:**
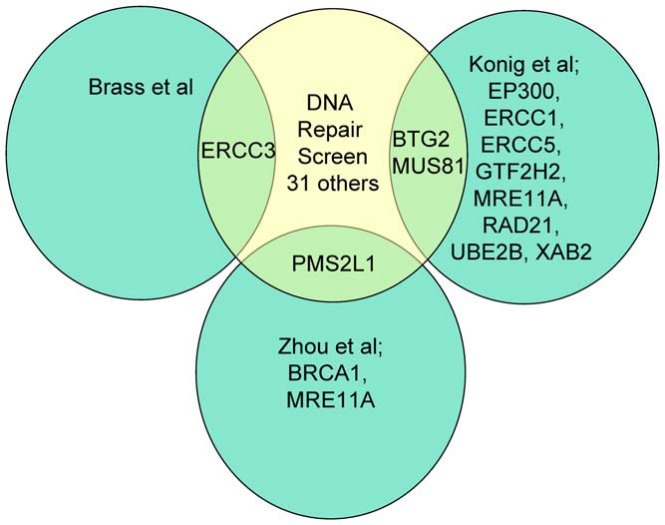
Venn Diagram of the DNA repair genes identified as important for HIV infection in three genome scale siRNA screens compared with this work.

Comparing our results with a targeted siRNA screen to those of genome scale screens reveals that although the genome scale screens all identified DNA repair factors, there was no overlap in the specific DNA repair factors identified, and no indication of which DNA repair pathway(s) may be most relevant for HIV replication. We identified ERCC3 [6], BTG2, MUS81 [7] and PMS2L1 [5] in common with the three previous genome scale screens (Figure 5). ERCC3, the single DNA repair factor included in our library that was identified by Brass et al [6] was also identified in our screen. Konig et al [7] identified ten DNA repair genes targeted in our siRNA library (BTG2, EP300, ERCC1, ERCC5, GTF2H2, MRE11A, MUS81, RAD21, UBE2B and XAB2). We also identified BTG2 and MUS81-targeting siRNAs as inhibitors of HIV replication, although we found that transfection of siRNAs targeting EP300, GTF2H2 and UBE2B enhanced HIV infection by at least two-fold, and we did not see a robust effect with siRNAs targeting any of the remaining genes. Comparing the screen reported here with our own genome scale screen [8], we previously reported a decrease in HIV infection following transfection with five siRNA pools targeting DNA repair enzymes, two of which were only effective at 96 h post-infection. Of the three remaining targeted mRNAs, (PMS2L1, MRE11A, and BRCA1), the pool targeting PMS2L1 was a strong inhibitor of HIV infection, as it was in the genome scale study, while neither the MRE11A nor the BRCA1 siRNA pools were effective. However; neither of the siRNA pools targeting MRE11A and BRCA1 used in this study included the same siRNA sequences used in the genome scale screen, and differences in siRNA efficacy may explain the discrepancy between the results. Comparing the data from the DNA repair focused screen to that generated in genome scale screens, it is clear that by focusing on a smaller set of genes in this targeted screen, we were able to reproduce some of the results from previous screens. Moreover, we observed more overlap with these screens than they had with each other, and we were able to extend the general observation that DNA repair is important to HIV infection by pinpointing a pathway of interest.

Somewhat surprisingly, the DNA repair pathway most frequently associated with HIV integration, the NHEJ DNA repair pathway, did not appear prominent in our screen. These results are consistent with those reported from previous genome scale siRNA screens for HIV host factors, as well as an shRNA screen carried out in a T-cell line, but inconsistent with a ribozyme-based screen in which Ku80 knockdown decreased both retroviral integration and tat-mediated transcription [5]–[7], [23]–[24]. The role of the NHEJ process in HIV replication is suggested to be repairing the gaps in chromosomal DNA left following integration or in circularizing unintegrated vDNA [25], [26], [10]–[12]. While this may be essential for host cell viability, it is not clear that it is essential for expression of retroviral genes, thus siRNA screens designed with tat-mediated expression of a reporter gene may not provide a reliable readout for factors involved in post-integration DNA repair.

Transfecting HeLa P4/R5 cells with siRNAs targeting15 BER genes reduced HIV infection in these cells by up to 70%, seemingly by decreasing vDNA integration. One possible role for BER enzymes in HIV integration may be in the repair of damaged vDNA if damaged DNA is poorly bound or processed by HIV integrase. An increase in the overall amount of damage in vDNA could be consistent with the high level of variability we observed when we measured vDNA levels to determine whether the BER siRNAs had an effect on reverse transcription, as vDNA damage would not be expected to be uniform and could have an effect on primer binding in the rtPCR assays used for detection. Alternatively, as HIV integration and BER protein complex formation have both been more frequently associated with open chromatin [27]–[29], a non-conflicting conjecture is that BER enzyme complexes could target the PIC to the host chromosome at sites of DNA damage.

This is the first report to identify a role for the short patch base excision repair pathway in HIV replication, although individual BER genes have been associated with HIV disease. HIV tat upregulates expression of POLB and OGG1 [30]–[31], suggesting the importance of these proteins for successful viral replication. Individuals infected with HIV exhibit increased levels of intracellular oxidative stress and oxidative damage as HIV infection progresses to AIDS [32]. siRNA-mediated knockdown of PARP1 was linked to a decrease in HIV integration [33]. Finally, siRNA-mediated knockdown of APEX1 was shown to decrease HIV infection [34].

In summary, we conducted an siRNA screen to identify DNA repair pathways important for HIV infection and identified BER as being highly represented among targets of effective siRNAs. We validated the role of 7 of the targeted BER genes in HIV replication by rescuing the siRNA-mediated knockdown with cDNA expression of the targeted gene, and extended the observation further by showing that NEIL1−/−, MUTYH−/−,OGG1−/−, and POLB−/− cell lines are less effectively transduced by HIV-based retroviral vectors. We explored the role BER plays in HIV replication, and showed that when BER is knocked down, HIV enters the cell, is reverse transcribed and trafficked to the nucleus, but vDNA integration is decreased. Further exploration of BER and its role in HIV replication could lead to the identification of new host factor targets for antiretroviral drugs.

## Methods

### Materials

HXB2 HIV was obtained from Advanced Biotechnologies (Columbia, MD). siRNAs used in the screen were designed using an algorithm developed to increase the targeting efficiency of the siRNA while reducing off-target silencing [35]. siRNAs were synthesized by Sigma-Proligo (The Woodlands, TX). The HIV integrase inhibitor, L-870,810 was obtained from the MRL compound collection [36].

### siRNA Screen

HeLa P4/R5 cells [37] were transfected with pools of 3 siRNAs (Table S1) as described in [5]. Briefly, cells were plated in 96-well plates and transfected with 100 nM of the siRNA pools using Oligofectamine (Invitrogen, Carlsbad, CA). Twenty-four hours following transfection, cells were infected with HXB2 HIV. Two uninfected wells of cells were included in each plate as a control for background levels of β-galactosidase activity. Two days after infection, β-galactosidase activity was assayed using Gal-Screen (Applied Biosystems, Carlsbad, CA) as per the manufacturer's instructions. Background measurements were subtracted from all wells, and β-galactosidase activity was normalized to the activity measured with the nonsilencing (luciferase) control siRNA. All transfections were conducted in duplicate. Cell viability following siRNA transfection was measured separately using Alamar Blue (Invitrogen, Carlsbad, CA) according to the manufacturer's instructions. siRNAs targeting Cyclin T1 and CDK9 were included as positive controls, while an siRNA targeting luciferase and a mock transfection were included as negative controls.

### cDNA Rescue

cDNAs encoding APEX1, LIG3, MUTYH, NEIL2, NTHL1, POLL and OGG1 were purchased from Invitrogen (Carlsbad, CA) and cloned into pcDNA3.1 (Invitrogen, Carlsbad, CA). siRNAs targeting the 3′ UTR of the gene are described in Table 2. siRNAs and the cDNA-expressing plasmid were co-transfected into HeLa P4/R5 cells using Lipofectamine 2000 (Invitrogen, Carlsbad, CA). Cells were infected and assayed as described [5].

### HIV Entry Assay

HeLa P4/R5 cells were transfected with siRNAs as described above and in [5]. HeLa P4/R5 cells were plated in 96 well plates and transfected with siRNAs using Oligofectamine (Invitrogen, Carlsbad, CA). 24 h following transfection, the entry of HIV virus-like particles was assayed. Media was removed and replaced with media containing HIV virus-like particles containing β-lactamase as described [20]. The virus-like particles were incubated with the transfected cells for 3 h at 37C. The cells were then washed with phenol red-free DMEM. CCF4-AM from GeneBlazer (Invitrogen, Carlsbad, CA) was added to each well to a final concentration of 5 µM. Cells were incubated with the CCF4-AM β-lactamase substrate overnight at room temperature in the dark. β-lactamase activity was measured the following day as an measurement of viral entry as it indicates the delivery of the contents of the virus-like particle into the cell. Activity was measured by reading fluorescence with excitation at 405 nm and emission at 460 nm (detects cleaved CCF4-AM substrate), or 535 nm (uncleaved CCF4-AM).

### HIV-Tat Assay

The assay for evaluating tat-mediated transactivation through the HIV-LTR was conducted as described [5]. HeLa P4/R5 cells were plated into 96-well plates and transfected with siRNAs at a final concentration of 100 nM using Oligofectamine (Invitrogen, Carlsbad, CA). The following day, the cells were transfected with pUCd5-Tat, an HIV1-tat expression vector, using Lipofectamine 2000 (Invitrogen, Carlsbad, CA) according to the manufacturer's instructions. The following day, β-galactosidase activity induced by the expression of HIV1-tat was detected using Lysis buffer plus substrate (25∶1) (Applied Biosystems) and measured using a VictorLight luminometer (PerkinElmer).

### qPCR analysis

For mRNA measurements, total cellular RNA was purified using RNeasy Plus (Qiagen, Valencia, CA) according to the manufacturer's instructions. mRNA levels were then determined by qrtPCR using primer and probe sets designed and synthesized by Applied Biosystems (Foster City, CA) and mRNA levels were normalized to cyclophilin.

For evaluating reverse transcription and integration of viral RNA: HeLa P4/R5 cells were plated in 6 well culture plates, and transfected with siRNA at a final concentration of 100 nM using Oligofectamine (Invitrogen, Carlsbad, CA) as described above and in [5]. The following day, the cells were infected with HXB2 HIV as described. DNA was extracted 48 hours after infection using DNeasy (Qiagen, Valencia, CA). HIV reverse transcribed or integrated vDNA was measured using Applied Biosystems 7500 Fast Real-Time PCR System and primer and probe sets essentially as described [21].

vDNA production was quantified using the 5NCR forward primer 5′GGCTAACTAGGGAACCCACTGCTT-3′, the 5NCR reverse primer 5′-AGCCGAGTCCTGCGTCG-3′, and the 5NCR probe 5′-(FAM)-CCTCAATAAAGCTTGCCTTGAGTGCTTCAA-(TAMRA)-3′. The qPCR reaction was initiated by 2 minutes at 50 C, followed by 95 C for 5 minutes, and then 45 cycles of 95 C for 15 seconds followed by 60 C for 1 minute.

Integrated vDNA was quantified using the Alu/LTR forward primer 5′-AACTAGGGAACCCACTGCTTAAG-3′, the Alu/LTR reverse primer 5′-TGCTGGGATTACAGGCGTGAG-3′ , and the Alu/LTR probe 5′-(FAM)-ACACTACTTGAAGCACTCAAGGCAAGCTTT-(TAMRA)-3′. The qPCR reaction was initiated by 2 minutes at 50 C, followed by 95 C for 5 minutes, and then 45 cycles of 95 C for 15 seconds followed by 60 C for 1 minute 30 seconds.

### HIV vector transduction of BER deletion cell lines

HIV vector particles expressing GFP following integration were generated by cotransfecting HEK293T (ATCC, Manassas, VA) cells with a VSVG envelope protein expression vector, a packaging vector, and a plasmid expressing genomic HIV RNA including a CMV promoter that drives expression of GFP as described [38]. At 72 hpi, cells were analyzed by flow cytometry for GFP expression. The retroviral particles were collected from the cell media, filtered to remove cells, and treated with DNAse to remove plasmid and cellular DNA. Mouse embryonic fibroblasts with deletions of Mutyh, Ogg1, Neil1, or PolB have been described [16]–[19]. Cells were infected with the retroviral vector, incubated for 72 h, fixed with paraformaldehyde, and analyzed for GFP expression by flow cytometry (BD FACS Calibur and CellQuest software). The relative level of GFP expressing cells was normalized to cells from wildtype littermates.

## Supporting Information

Table S1 **Data from the targeted HIV infectivity screen.** Transcript ID refers to the accession number for the mRNA transcript against which the siRNAs were designed. The Gene Name column contains the gene symbol for the targeted gene. The “Pool siRNA #1 sense sequence”, “Pool siRNA #2 sense sequence”, and “Pool siRNA #3 sense sequence” columns contain the sense strand sequences for each of the siRNAs in the pool used in the screen. The “Average activity (% of lucif)” column contains the HIV infectivity data for HeLa P4/R5 cells transfected with the indicated siRNA pool normalized to the two nonsilencing (luciferase-targeting) controls included in the same 96-well plate. The average of two separate experiments is shown. The final column, “siRNA Toxicity” indicates whether cell toxicity was observed in parallel transfections with the siRNA pool. siRNA pools causing toxicity are indicated with a “YES” in this column. siRNAs that caused toxicity and those that did not decrease HIV infectivity by more than 40% were not studied further, and this is indicated by shading these rows gray.
(XLS)

Table S2 **Key to assignment of hits in** **Figure 1B** **.** Hits assigned to BER, NER, NHEJ, DSB, MMR, HR, SSBR, or other from the primary screen are listed. Additional BER enzymes for which two or more effective single siRNAs were identified are listed in italics below the list from the primary screen.
(XLS)
